# Diverse Genomic Mechanisms of *cfiA* Activation in Carbapenem-Resistant *Bacteroides fragilis* Clinical Isolates from Korea

**DOI:** 10.3390/antibiotics15080763

**Published:** 2026-08-09

**Authors:** Yangsoon Lee, Hyukmin Lee, Yoon Soo Park, Myungsook Kim, Kyungwon Lee

**Affiliations:** 1 Department of Laboratory Medicine, Hanyang University College of Medicine, Seoul 04763, Republic of Korea; 2 Department of Laboratory Medicine, Research Institute of Bacterial Resistance, Yonsei University College of Medicine, Seoul 03722, Republic of Korea; 3 Division of Infectious Disease, Department of Internal Medicine, Yongin Severance Hospital, Yonsei University College of Medicine, Yongin 16995, Republic of Korea; 4 Infectious Disease Research Center, Seoul Clinical Laboratories, Yongin 16954, Republic of Korea

**Keywords:** *Bacteroides fragilis*, carbapenem resistance, *cfiA*, insertion sequence, promoter duplication, whole-genome sequencing

## Abstract

**Background:** *Bacteroides fragilis* is the most clinically important anaerobic bacterium and has shown increasing carbapenem resistance mediated by the *cfiA* metallo-β-lactamase. Although insertion sequence (IS)-mediated activation of *cfiA* is a well-recognized mechanism, the contribution of IS-independent genomic mechanisms remains incompletely characterized. This study investigated the phenotypic and genomic mechanisms of *cfiA*-associated carbapenem resistance in clinical *B. fragilis* isolates from Korea. **Methods:** Twenty-six non-duplicate carbapenem-nonsusceptible *B. fragilis* isolates were collected from three Korean institutions between 2022 and 2023. Antimicrobial susceptibility was tested by disk diffusion and agar dilution methods, and carbapenemase activity was assessed using the anaerobic carbapenem inactivation method (Ana-CIM) and the meropenem double-ended Etest. Whole-genome sequencing was performed to identify resistance determinants, insertion sequences, promoter structures, and multilocus sequence types. **Results:** Among 26 isolates, 12 (46.2%) were *cfiA*-positive and 14 (53.8%) were *cfiA*-negative. High-level resistance (imipenem MIC ≥ 32 mg/L; meropenem MIC ≥ 128 mg/L) was observed in four *cfiA*-positive isolates carrying activating IS elements (IS1187 or IS942) upstream of *cfiA*. Notably, three additional highly resistant *cfiA*-positive isolates lacked upstream IS elements and showed distinct genomic features, including tandem duplication of *cfiA2* (SEV23_101), duplicated promoter architecture upstream of *cfiA14* (SEV23_15), and high-level resistance associated with *cfiA18* (SCL22_40). Ana-CIM yielded positive results only among isolates with meropenem MIC ≥ 128 mg/L, whereas the meropenem double-ended Etest failed to discriminate *cfiA*-associated carbapenemase activity. **Conclusions**: These findings indicate that high-level carbapenem resistance in *B. fragilis* may be associated with several genomic features, including *IS*-mediated activation, increased *cfiA* gene dosage, and promoter amplification. Molecular characterization is therefore essential for accurate interpretation of carbapenem resistance in *B. fragilis*.

## 1. Introduction

*Bacteroides fragilis* is one of the most clinically important anaerobic Gram-negative bacteria and is frequently isolated from intra-abdominal infections, bloodstream infections, abscesses, and other deep-seated infections [1,2,3]. The *B. fragilis* group is generally susceptible to metronidazole, carbapenems, chloramphenicol, and β-lactam/β-lactamase inhibitor combinations, making these agents the cornerstone of treatment [4]. Among these, carbapenems are regarded as the most effective agents against *B. fragilis* strains exhibiting high-level resistance to other antibiotics [4]. However, *Bacteroides* bacteremia has been linked to higher mortality rates, which can be further worsened by the use of inactive antimicrobials in the treatment regimen [5]. Rising carbapenem resistance has been associated with increased 30-day mortality [6].

As carbapenem resistance mechanisms in *B. fragilis* are varied, there is currently no consensus on the optimal empirical antibiotic choice for infections caused by this organism [7]. These clinical consequences underscore the urgent need to comprehensively understand the full spectrum of carbapenem resistance mechanisms in *B. fragilis*, including IS-independent pathways, and to improve laboratory recognition of clinically significant resistance phenotypes for appropriate therapeutic decision-making and antimicrobial stewardship. Carbapenem resistance in *B. fragilis* is most commonly associated with the chromosomal metallo-β-lactamase gene *cfiA*. *B. fragilis* is genetically divided into two major divisions: division I strains, which are *cfiA*-negative, and division II strains, which harbor *cfiA* [4,8]. However, the presence of *cfiA* alone does not necessarily result in carbapenem resistance. In many *cfiA*-positive isolates, the gene remains transcriptionally silent or weakly expressed, leading to susceptibility or only low-level resistance in routine antimicrobial susceptibility testing [4,9]. High-level carbapenem resistance is typically associated with insertion sequence (IS) elements located upstream of *cfiA*, which provide strong promoter activity and enhance *cfiA* expression [4,9,10]. The genotype–phenotype relationship is nevertheless more complex than this classical model suggests. IS-less *cfiA*-positive isolates with elevated carbapenem MICs have been described, while *cfiA*-negative isolates may also exhibit carbapenem nonsusceptibility through alternative enzymatic or non-enzymatic mechanisms. Potential IS-independent pathways include intrinsic promoter activity, promoter rearrangement, increased *cfiA* copy number, altered permeability, and efflux-related mechanisms [11,12]. A detailed genomic analysis is therefore required to explain isolates that do not conform to the conventional IS-mediated activation model.

Phenotypic antimicrobial susceptibility testing may not reliably distinguish *cfiA*-positive from *cfiA*-negative isolates or determine whether *cfiA* is functionally activated. Several methods, including matrix-assisted laser desorption/ionization time-of-flight mass spectrometry (MALDI-TOF MS)-based prediction, the anaerobic carbapenem inactivation method (Ana-CIM), and inhibitor-based tests such as the meropenem double-ended Etest [13], have been proposed to detect *cfiA* carriage or carbapenemase activity. However, the performance and clinical utility of these approaches may vary depending on the level of *cfiA* expression and the underlying genomic mechanism of resistance. This study aimed to characterize the phenotypic and genomic features of carbapenem-nonsusceptible *B. fragilis* clinical isolates collected from three institutions in Korea. In particular, we investigated both IS-mediated and IS-independent mechanisms associated with *cfiA* activation to improve the interpretation of carbapenem resistance in clinical isolates.

## 2. Results

### 2.1. B. fragilis Isolates and Antimicrobial Susceptibility Testing

All isolates were nonsusceptible to meropenem or imipenem based on disk diffusion inhibition zone diameters or agar dilution MICs (Table 1). Twelve isolates belonged to division II *B. fragilis* and carried the *cfiA* gene as confirmed by whole-genome sequencing (WGS; referred to as *cfiA*-positive), whereas 14 belonged to division I and lacked *cfiA* (*cfiA*-negative). Of the 12 division II isolates, seven (58.3%) exhibited high-level resistance, with meropenem MICs ranging from 128 mg/L to >256 mg/L and imipenem MICs ranging from 32 mg/L to 128 mg/L. The resistance phenotype was heterogeneous. Among *cfiA*-positive division II isolates, two isolates had meropenem MICs of only 4 mg/L. Conversely, two *cfiA*-negative division I isolates showed meropenem MICs ≥ 16 mg/L.

### 2.2. MBL Activity Test Using MALDI-TOF MS, Ana-CIM and the Meropenem Double-Ended Etest

MALDI-TOF MS correctly predicted *cfiA* carriage in all 12 *cfiA*-positive isolates but yielded one false-positive result (SEV23_85), resulting in an overall concordance of 96.2% (25/26) with WGS. Among the 12 division II isolates, seven (58.3%) with high-level carbapenem resistance tested positive by Ana-CIM, showing inhibition zone diameters of 6 mm. The remaining five division II isolates and all division I isolates tested negative by Ana-CIM. Thus, Ana-CIM positivity correlated more closely with high-level carbapenem resistance than with *cfiA* carriage alone. The meropenem double-ended Etest showed poor discriminatory ability for *cfiA*-associated carbapenemase activity, even after two independent tests. Using the manufacturer’s criterion of at least a threefold reduction in the MIC to define carbapenemase positivity, 25 of the 26 (96.2%) isolates were interpreted as positive (Table 1).

### 2.3. Antimicrobial Resistance-Associated Genes

**Figure 1:**
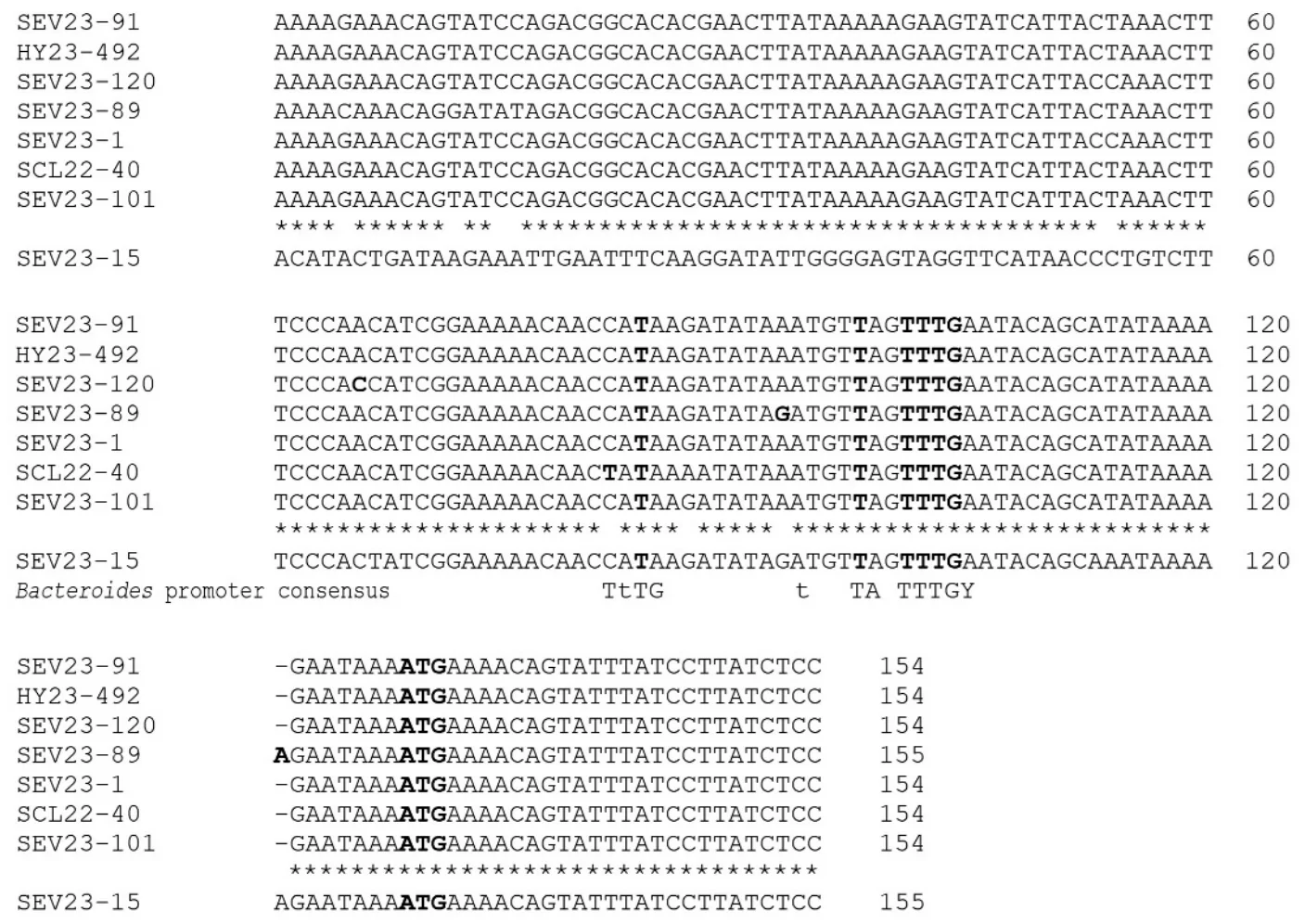
ClustalW alignment of upstream regions of *cfiA* genes in IS-less *B. fragilis* isolates. Matching nucleotides are indicated by stars below the sequences. Strain names are shown on the left. The start codon and nucleotides matching the *Bacteroides* promoter consensus are in bold.

**Figure 2:**
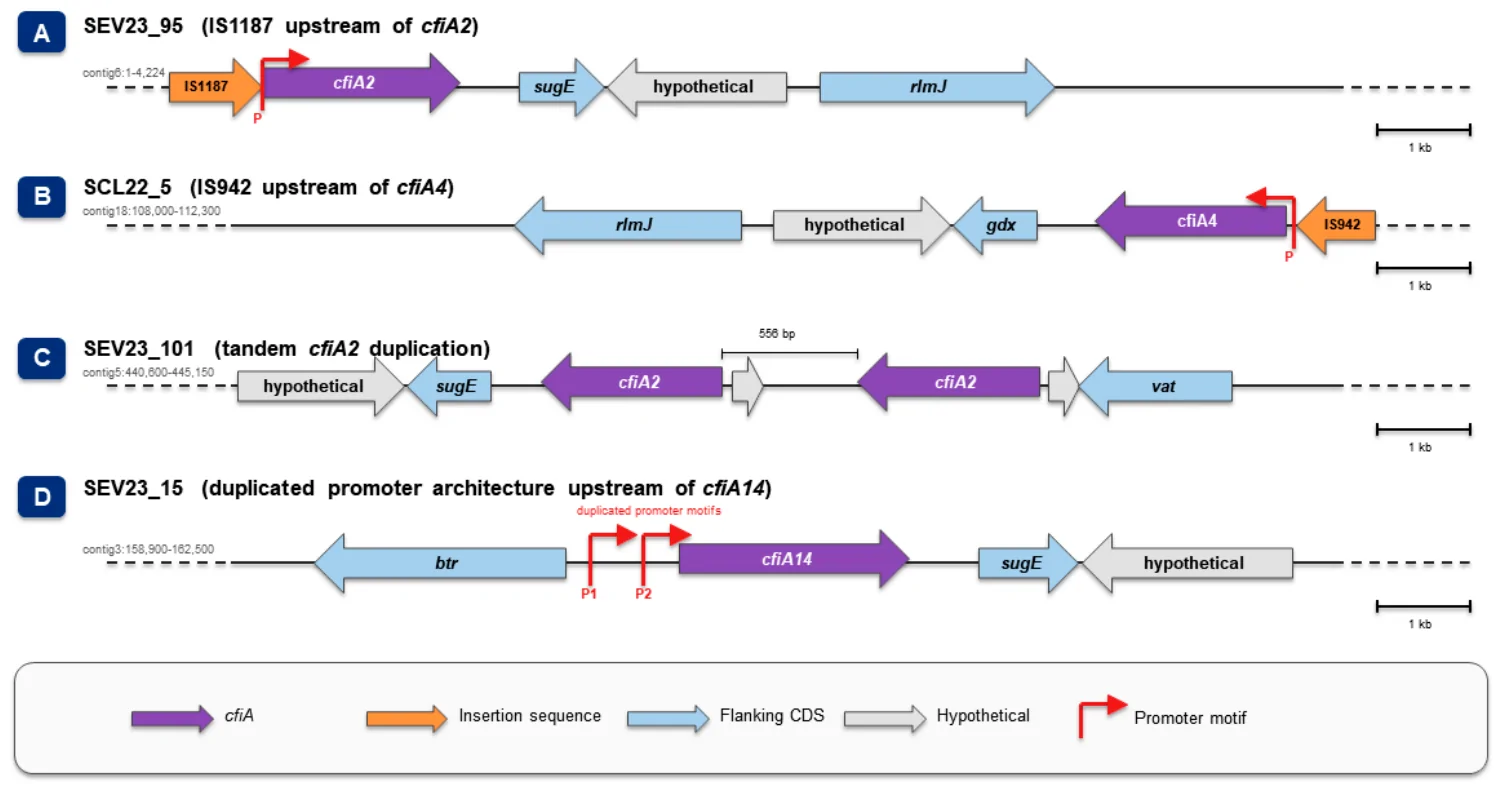
Genomic structures surrounding *cfiA* in representative carbapenem-resistant *B. fragilis* isolates. (**A**) IS1187 located upstream of *cfiA2* in SEV23_95. (**B**) IS942 located upstream of *cfiA4* in SCL22_5. (**C**) Tandem duplication of *cfiA2* in SEV23_101. (**D**) Duplicated promoter architecture upstream of *cfiA14* in SEV23_15. Arrows indicate coding sequences, and promoter motifs are indicated by red arrows. The figure illustrates both IS-mediated and IS-independent genomic mechanisms associated with *cfiA* activation.

MLST revealed substantial genetic diversity among the isolates (Table 1). In the *cfiA*-positive group, ST157 was the most common sequence type, detected in four of 12 isolates (33.3%). Among the 12 *cfiA*-positive isolates, four isolates carrying IS elements, including IS1187 or IS942 upstream of *cfiA*, exhibited high meropenem MICs ranging from 128 mg/L to >256 mg/L. The remaining *cfiA*-positive isolates did not possess IS elements upstream of *cfiA*, but nucleotide sequences matching the *Bacteroides* promoter consensus were detected (Figure 1). Among the *cfiA*-positive isolates, three highly resistant strains lacked IS upstream of *cfiA*. SEV23_101 contained two tandem copies of *cfiA2* and exhibited high-level resistance (imipenem MIC, 64 mg/L; meropenem MIC, >256 mg/L). SEV23_15 carried *cfiA14* and displayed two putative promoter-like motifs upstream of *cfiA*, consistent with the markedly elevated carbapenem MICs observed in this isolate (imipenem MIC, 128 mg/L; meropenem MIC, 256 mg/L). SCL22_40, carrying *cfiA18*, also showed high-level resistance despite the absence of upstream IS elements or apparent promoter duplication (Figure 2). The *cfiA18* allele was assigned based on the CARD/NCBI reference sequence (CARD ARO:3006910; GenBank accession no. NG_054673.1; protein accession no. WP_085562388.1).

## 3. Discussion

The most important finding of this study is that high-level carbapenem resistance in *B. fragilis* was not exclusively associated with IS-mediated activation of *cfiA*. In *cfiA*-positive isolates, intrinsic promoter activity, promoter rearrangement, or increased *cfiA* copy number may contribute to increased *cfiA* expression, whereas *cfiA*-negative isolates may rely on alternative, non-*cfiA*-mediated resistance mechanisms. Although the conserved promoter sequence was shared among the IS-less *cfiA*-positive isolates, their meropenem MICs varied widely from 4 to >256 mg/L. This finding indicates that the conserved promoter sequence alone does not explain the resistance phenotype and suggests that additional factors, such as regulatory or genomic context, may also contribute to the resistance phenotype. Therefore, a detailed genomic investigation is needed to clarify the mechanisms underlying carbapenem nonsusceptibility, particularly in isolates that are not consistent with the classical IS-mediated *cfiA* activation model. Although IS1187 and IS942 were strongly associated with elevated carbapenem MICs as shown in previous studies [14,15], several highly resistant isolates lacked activating IS elements in this study. Genomic analysis revealed diverse architectures, including tandem duplication of *cfiA* and duplicated promoter motifs, suggesting that increased gene dosage and promoter duplication may represent underrecognized genomic mechanisms potentially associated with *cfiA* activation. The higher *cfiA* copy number observed in SEV23_101 provides additional support for the gene-dosage hypothesis, although transcriptional and functional validation is still required. In contrast, SCL22_40 carried neither an activating IS element nor *cfiA* duplication, and the functional significance of the *cfiA18*/D26N variant remains unknown. Therefore, the mechanism underlying its high-level carbapenem resistance remains unresolved. Together, these observations suggest that *cfiA* expression may be regulated by mechanisms beyond the classical IS-mediated model, although further validation is required. Although ‘IS-less’ *B. fragilis* isolates exhibiting high-level carbapenem resistance have been previously reported in Europe and China by Sóki et al. [14] and Wang et al. [8] respectively, the underlying genomic mechanisms responsible for these phenotypes have remained largely uncharacterized. Our study provides molecular evidence that IS-independent mechanisms may contribute to *cfiA* activation by demonstrating *cfiA* tandem duplication and promoter architecture duplication. However, these findings provide genomic observations that may provide a basis for IS-independent resistance, but additional expression, copy-number, and functional studies are needed to determine whether the identified genomic features directly contribute to carbapenem resistance.

The heterogeneous relationship between genotype and phenotype has important diagnostic implications. Some *cfiA*-positive isolates can remain susceptible or show only low-level resistance when *cfiA* is not efficiently expressed, whereas some *cfiA*-negative isolates can show nonsusceptible phenotypes through other mechanisms such as the overexpression of RND-family multidrug efflux pumps and loss or alteration of outer membrane proteins (OMPs) [1,4]. Because our study was based on WGS, these mechanisms could not be evaluated directly. WGS alone cannot determine expression-level changes such as OMP loss or increased efflux pump activity. Since January 2022, EUCAST has classified *B. fragilis* isolates with meropenem MIC ≤ 1 mg/L as susceptible, a change intended to reduce underestimation of *cfiA*-positive strains [16]. However, our results indicate that breakpoint-based categorization alone cannot accurately infer *cfiA* carriage or activation status. Therefore, carbapenemase detection tests such as Ana-CIM, MALDI-TOF MS-based prediction, and molecular methods may provide complementary information for antimicrobial stewardship.

AST results obtained by disk diffusion test (DDT) and agar dilution method (ADM) did not consistently reflect the presence or activation status of *cfiA*. Therefore, additional methods for detecting *cfiA* carriage or carbapenemase activity may be useful for interpreting resistance mechanisms and supporting antimicrobial stewardship. MALDI-TOF MS does not directly detect the *cfiA* gene. Instead, it differentiates *cfiA*-positive and cfiA-negative *B. fragilis* isolates based on characteristic differences in their protein mass spectra, reflecting the distinct phylogenetic lineages of division II and division I isolates, respectively [17,18]. It is a simple and rapid tool for predicting the presence of *cfiA*, but false positives can occur. In this study, SEV23_85 was identified as *cfiA*-positive by MALDI-TOF MS, whereas WGS did not detect *cfiA*, supporting the interpretation of a false-positive spectral classification. The Bruker Biotyper relies on protein mass patterns, which can overlap between *cfiA*-positive and *cfiA*-negative strains. Several studies have suggested that MALDI-TOF MS-based prediction of *cfiA* is useful for screening but requires confirmatory genetic testing for clinical interpretation [17,18].

Ana-CIM was developed to improve detection of carbapenemase activity in *B. fragilis* [19]. In the present study, Ana-CIM was positive only when the meropenem MIC was ≥128 mg/L, whereas all isolates with MICs ≤ 32 mg/L tested negative. These results suggest that Ana-CIM primarily detects phenotypically significant carbapenemase activity associated with high-level resistance rather than the mere presence of *cfiA*. In contrast, the meropenem double-ended Etest did not reliably differentiate MBL-producing isolates in this collection, limiting its usefulness for detecting *cfiA*-mediated resistance in *B. fragilis*. Strains harboring both an upstream IS element and *cfiA* exhibited high-level carbapenem resistance, supporting the established role of IS-mediated promoter activation. IS1187 and IS942 were associated with markedly elevated carbapenem MICs and likely drive high transcriptional activity of *cfiA* [14]. However, the IS-negative high-level resistant isolates were particularly noteworthy. SEV23_101 showed tandem *cfiA2* duplication, which may increase gene dosage. SEV23_15 showed a distinct upstream sequence with two predicted promoter-like motifs, suggesting promoter duplication as an alternative activation mechanism. SCL22_40 carried *cfiA18* without upstream IS elements and showed high-level resistance; because *cfiA18* differs from *cfiA1* by a D26N substitution, variant-dependent differences in enzyme activity or expression cannot be excluded. However, the contribution of this substitution to resistance remains unclear because enzyme activity, gene expression, and functional effects were not evaluated. These findings support the concept that high-level resistance can emerge through both IS-dependent and IS-independent mechanisms. In addition, MLST revealed substantial genetic diversity among the isolates, and the presence of multiple sequence types among *cfiA*-positive isolates suggests that carbapenem resistance was not attributable to a single clonal lineage.

Our study identified three *cfiA*-negative division I isolates—SCL23_30 (meropenem MIC 32 mg/L), SCL22_13 (imipenem MIC 32 mg/L), and SEV23_98 (meropenem MIC 16 mg/L)—that exhibited carbapenem resistance. Notably, WGS revealed that SCL23_30 and SCL22_13 harbored the class D β-lactamase gene, *bla*_OXA-347_, in addition to the endogenous *cepA* gene. Although *cepA* is typically associated only with resistance to penicillins and early-generation cephalosporins, acquired *bla*_OXA-347_ has been reported to confer meropenem resistance upon overexpression, representing an alternative enzymatic pathway for carbapenem nonsusceptibility in division I strains [20,21]. In addition, the *cfiA*-positive division II isolate SCL22_5 carried both *cfiA* and *bla*_OXA-347_, making it difficult to attribute its carbapenem resistance phenotype solely to *cfiA*. For isolates such as SEV23_98, which lacked both *cfiA* and *bla_OXA-347_* but still showed nonsusceptibility to meropenem or imipenem, the resistance phenotype may stem from non-enzymatic mechanisms or currently uncharacterized resistance determinants not represented in existing resistance gene databases. We examined the existing draft genome assembly of SEV23_98 for sequence-level abnormalities in efflux pump and outer-membrane protein loci. Multiple relevant loci, including *bepE*, *bepF*, *oprM*, *mexB*, *mdt*, *tolC*, and *ompA*, were identified. No obvious gene loss, frameshift annotation, premature termination, pseudogenization, or gross truncation was detected. Although several nucleotide substitutions were observed, their functional significance could not be determined without expression or functional analyses. Further transcriptomic, comparative genomic, and functional studies are required to identify the underlying resistance mechanism. Importantly, each proposed IS-independent mechanism in this study was observed in only a single isolate (n = 1), which substantially limits the strength and generalizability of these interpretations.

This study has several limitations. First, the number of isolates was limited, and the findings require validation in larger collections. Second, *cfiA* expression was not quantified by transcriptional analysis, and the functional effects of promoter duplication, *cfiA* tandem duplication, and the *cfiA18* variant were not confirmed experimentally. Third, non-*cfiA*-mediated mechanisms in division I isolates were inferred from genomic data and require further transcriptomic or functional validation. Fourth, because the cohort was preselected to include only carbapenem-resistant isolates, the true prevalence or frequency of these mechanisms could not be estimated.

## 4. Materials and Methods

### 4.1. Bacterial Isolates and MALDI-TOF MS Analysis

A total of 26 non-duplicate clinical isolates of carbapenem-nonsusceptible *B. fragilis* were collected from Hanyang University Hospital, Severance Hospital and Seoul Clinical Laboratories in Korea, between January 2022 and December 2023. Isolates were obtained from 9 blood, 9 abscess specimens, and 8 body fluid specimens submitted for routine clinical culture. Only one isolate per patient was included. Species identification was performed using MALDI-TOF MS Biotyper (Bruker Daltonics, Bremen, Germany). The instrument-generated classification used for prediction of *cfiA*-associated division II status was recorded and subsequently compared with WGS-based *cfiA* detection. Isolates were subcultured under anaerobic conditions before phenotypic and genomic testing.

### 4.2. Antimicrobial Susceptibility Tests Using DDT and ADM

DDT was performed according to EUCAST guidelines [16,22]. Fastidious anaerobe agar (MB Cell, Seoul, Republic of Korea) supplemented with 5% defibrinated horse blood (FAA-HB) was used as the culture medium. Bacterial suspensions were prepared in normal saline to a turbidity equivalent to a 1.0 McFarland standard. Imipenem (10 μg) and meropenem (10 μg) antimicrobial disks (Oxoid, Hampshire, UK) were used. The plates were incubated in an anaerobic chamber (Bactron, Cornelius, OR, USA) at 35 °C for 16–20 h.

ADM was conducted in accordance with CLSI guidelines [23]. Brucella agar supplemented with hemin and vitamin K_1_ (Sigma-Aldrich, Seoul, Republic of Korea) and 5% laked sheep blood was used as the culture medium. Antimicrobial powders were imipenem and meropenem (Sigma-Aldrich). An inoculum of 10^5^ colony-forming units (CFU) was applied using a Steers replicator (CMI-Promex Inc., Pedricktown, NJ, USA), and the plates were incubated in an anaerobic chamber at 35 °C for 48 h. *B. fragilis* ATCC 25285 and *B. thetaiotaomicron* ATCC 29741 were used as quality control strains.

### 4.3. Anaerobic Carbapenem Inactivation Method

Ana-CIM was performed twice as previously described [19]. Isolates were subcultured on Brucella blood agar supplemented with hemin and vitamin K_1_ (Asan Medical, Gimpo, Republic of Korea), with a 10 μg meropenem disk placed in the first quadrant, and incubated anaerobically at 35 °C for 24–48 h. A fresh meropenem disk was then incubated in 5 mL of Brucella broth at room temperature for 15 min to allow diffusion. Bacterial growth near the disk was suspended in meropenem-containing broth to achieve a suspension of 1.0 McFarland, followed by anaerobic incubation at 35 °C for 6 h. After incubation, the meropenem disk was transferred onto a Mueller–Hinton agar plate inoculated with a 0.5 McFarland suspension of *E. coli* ATCC 25922 and incubated aerobically at 35 °C for 18 h. Inhibition zones were measured and interpreted as ≤8 mm for positive and ≥15 mm for negative results according to previous study criteria [19], with microcolonies inside the inhibition zones considered positive for carbapenemase activity. Ana-CIM results were interpreted in a blinded manner without knowledge of the isolates’ genotypes.

### 4.4. Meropenem Double-Ended Etest

Metallo-β-lactamase production was evaluated twice using the Etest MBL (bioMérieux, Marcy-l’Étoile, France), which consists of a meropenem strip and a meropenem–EDTA strip [13]. All tests were performed on Brucella blood agar supplemented with hemin and vitamin K_1_, using a 1.0 McFarland suspension prepared in normal saline. Plates were incubated anaerobically at 35 °C for 24 h. MICs were interpreted according to the manufacturer’s instructions. A ≥3-fold reduction in meropenem MIC in the presence of EDTA was considered indicative of MBL activity, in accordance with established criteria.

### 4.5. Whole-Genome Sequence Analysis

Bacterial isolates grown on Brucella blood agar were submitted to Macrogen (Seoul, Republic of Korea) for WGS. Genomic DNA libraries were prepared using the TruSeq Nano DNA Library Preparation Kit (Illumina, San Diego, CA, USA) with a target insert size of approximately 350 bp. Whole-genome sequencing was performed on an Illumina NovaSeq 6000 platform using 2 × 150 bp paired-end reads. Raw-read quality was assessed using FastQC v0.11.7, and adapter trimming and quality filtering were performed using Trimmomatic v0.38. Reads were retained when at least 90% of their bases had Phred quality scores of ≥20. De novo assembly was performed using multiple k-mer sizes with SPAdes v3.15.0. Assembly quality was assessed by read self-mapping, BLASTN, average nucleotide identity analysis, and BUSCO v5.1.3 using the bacteria_odb10 lineage dataset. The sequencing depth was approximately 100–150×. Across the 26 isolates, the median number of contigs was 34 (range, 9–107), the median assembly size was 5,399,531 bp (range, 4,824,350–5,631,928 bp), and the median N50 was 378,305 bp (range, 92,523–1,377,055 bp). The draft whole-genome sequences of all 26 *B. fragilis* isolates were submitted to NCBI under BioProject accession number PRJNA1499346. Sequence reads were assembled de novo, and assemblies were examined for species identity, multilocus sequence type (MLST), antimicrobial resistance genes, *cfiA* alleles, and the genomic regions surrounding *cfiA*. MLST and resistance-gene repertoires were determined using resources from the Center for Genomic Epidemiology (http://www.genomicepidemiology.org/) and PubMLST (https://pubmlst.org/) accessed in February 2026. Antimicrobial resistance genes were identified using ResFinder version 4.7.2 with the default settings. The minimum sequence identity threshold for resistance gene detection was set at 80%. IS elements were identified by comparison with ISfinder (https://www-is.biotoul.fr/index.php, accessed on 1 February 2026). For *cfiA*-positive isolates lacking an upstream IS element, upstream sequences were aligned and inspected for motifs matching the *Bacteroides* promoter consensus. The upstream regions of *cfiA* were aligned using ClustalW and manually compared with the previously reported *Bacteroides* promoter consensus sequence [12]. Putative promoter regions were further assessed using the BPROM bacterial promoter prediction program (Softberry, Inc., Mount Kisco, NY, USA; accessed in February 2026). Promoter-like motifs were identified when the upstream sequence showed similarity to the conserved −35 and −10 elements with an appropriate spacer length and was also predicted as a bacterial promoter by BPROM. Gene arrangement and copy number in representative isolates were evaluated from assembled contigs and used to construct the genomic schematics shown in Figure 2. For SEV23_101, the tandem *cfiA* arrangement was further evaluated by remapping the quality-filtered paired-end reads to the assembled contig using BWA. The two identical *cfiA* coding sequences were separated by a 556 bp inter-copy region. The arrangement was supported by 141 high-quality reads spanning the boundary between the first *cfiA* copy and the inter-copy region and 213 reads spanning the boundary between the inter-copy region and the second copy. All supporting reads had mapping quality scores of ≥20 and aligned continuously across the respective boundaries without clipping or alignment gaps. The genomic findings were interpreted together with agar dilution MICs and phenotypic carbapenemase-test results.

### 4.6. Data Analysis

Results were summarized descriptively because of the limited sample size and the exploratory design. Categorical findings are presented as numbers and percentages, and MICs and inhibition zone diameters are reported for individual isolates. Concordance between MALDI-TOF MS-based *cfiA* prediction and WGS was calculated as the proportion of isolates with matching results. Phenotypic findings were compared qualitatively with *cfiA* carriage, upstream IS elements, promoter architecture, and other genomic determinants.

## 5. Conclusions

High-level carbapenem resistance in *B. fragilis* was associated with IS-mediated activation of *cfiA* in some isolates, whereas several isolates showed IS-independent genomic features, including tandem *cfiA* duplication and duplicated promoter architecture. However, each proposed IS-independent mechanism was observed in only a single isolate, and no transcriptional or functional validation was performed. Therefore, these findings should be regarded as potential associations rather than established resistance mechanisms. Phenotypic methods alone did not reliably predict *cfiA* carriage or activation status. A combined strategy incorporating routine susceptibility testing, rapid screening, and targeted molecular confirmation may improve interpretation in clinical laboratories. Larger multicenter studies and functional analyses are required to determine the prevalence, expression effects, and clinical significance of these non-classical genomic features.

## Figures and Tables

**Table 1:** Antimicrobial susceptibility and genetic determinants of carbapenem resistance in 26 *Bacteroides fragilis* clinical isolates.

Strains	Disk Diffusion, Zone Diameter (mm)		Agar Dilution, MIC (mg/L)		MALDI-TOF MS	Ana-CIM (mm)	Meropenem Double-Ended Etest (mg/L)		Whole-Genome Sequence Analysis				
	IMP	MEM	IMP	MEM	*cfiA* Detection		MEM	MEMI	*cfiA* Type	IS Upstream of *cfiA*	*cepA* Type	*cfxA/OXA-347*	MLST
SEV23_95	19	6	64	256	+	6	>8	0.032	*cfiA2*	IS1187	-	-	157
SEV23_93	19	6	32	256	+	6	>8	0.032	*cfiA2*	IS1187	-	-	157
HY23_168	24	6	64	128	+	6	>8	0.032	*cfiA2*	IS1187	-	-	157
SCL22_5	6	6	128	128	+	6	>8	<0.032	*cfiA4*	IS942	-	*OXA-347*	151
SEV23_101	22	6	64	>256	+	6	>8	0.032	*cfiA2 + cfiA2*	-	-	-	165
SEV23_15	27	6	128	256	+	6	>8	<0.032	*cfiA14*	-	-	-	49
SCL22_40	28	6	32	128	+	6	>8	0.032	*cfiA18*	-	-	-	189
SEV23_91	31	19	4	16	+	21	>8	0.032	*cfiA2*	-	-	*cfxA*	157
SEV23_89	36	19	2	8	+	25	>8	0.032	*cfiA4*	-	-	*-*	342
HY23_492	33	21	4	8	+	20	>8	0.032	*cfiA32*	-	-	*-*	122
SEV23_1	33	19	1	4	+	20	>8	<0.032	*cfiA31*	-	-	*-*	343
SEV23_120	31	25	2	4	+	22	1.5	0.032	*cfiA2*	-	-	*cfxA*	344
SCL23_30	13	6	8	32	-	22	>8	0.032	-	-	*cepA*	*OXA-347*	170
SEV23_98	25	16	4	16	-	25	>8	0.032	-	-	*cepA44*	*-*	345
SEV23_38	29	27	4	8	-	25	>8	<0.032	-	-	*cepA*	*cfxA3*	1
SEV23_85	26	16	4	8	+	21	>8	0.032	-	-	*cepA*	*cfxA5*	1
SEV23_27	27	21	2	4	-	26	3	0.032	-	-	*cepA*	*cfxA4*	135
SEV23_41	26	22	2	4	-	25	2	<0.032	-	-	*cepA49*	*cfxA3*	11
SEV23_71	24	21	2	4	-	26	2	0.032	-	-	*cepA*	*cfxA5*	21
SEV23_78	26	22	2	4	-	26	2	0.032	-	-	*cepA*	*cfxA5*	1
SCL22_13	11	26	32	4	-	25	2	2	-	-	*cepA44*	*OXA-347*	144
SEV23_20	27	20	2	2	-	24	1.5	<0.032	-	-	*cepA*	*cfxA3*	135
SEV23_28	25	21	4	2	-	25	2	<0.032	-	-	*cepA49*	*cfxA3*	346
SEV23_100	26	22	2	2	-	25	1.5	0.064	-	-	*cepA29*	*-*	9
SCL22_34	12	27	8	2	-	25	4	0.032	-	-	*cepA44*	*cfxA5*	169
SEV23_96	19	25	4	1	-	25	0.38	0.047	-	-	*cepA*	*cfxA4*	143

Abbreviations: MALDI-TOF MS, matrix-assisted laser desorption/ionization time-of-flight mass spectrometry; Ana-CIM, anaerobic carbapenem inactivation method; IMP, imipenem; MEM, meropenem; MEMI, meropenem-inhibitor; -, not detected; MLST, multilocus sequence typing.

## Data Availability

The original contributions presented in this study are included in the article. Further inquiries can be directed to the corresponding author. The draft whole-genome sequence data for all 26 *Bacteroides fragilis* isolates are available in the NCBI database under BioProject accession number PRJNA1499346.

## References

[1] Dubreuil L.J. (2024). Fifty years devoted to anaerobes: Historical, lessons, and highlights. Eur. J. Clin. Microbiol. Infect. Dis..

[2] Fang H., Li X., Yan M.K., Tong M.K., Chow K.H., Cheng V.C., Ho P.L. (2023). Antimicrobial susceptibility of *Bacteroides fragilis* group organisms in Hong Kong, 2020–2021. Anaerobe.

[3] Nagy E., Boyanova L., Justesen U.S., ESCMID Study Group of Anaerobic Infections (2018). How to isolate, identify and determine antimicrobial susceptibility of anaerobic bacteria in routine laboratories. Clin. Microbiol. Infect..

[4] Yekani M., Rezaee M.A., Beheshtirouy S., Baghi H.B., Bazmani A., Farzinazar A., Memar M.Y., Sóki J. (2022). Carbapenem resistance in *Bacteroides fragilis*: A review of molecular mechanisms. Anaerobe.

[5] Nguyen M.H., Yu V.L., Morris A.J., McDermott L., Wagener M.W., Harrell L., Snydman D.R. (2000). Antimicrobial resistance and clinical outcome of *Bacteroides* bacteremia: Findings of a multicenter prospective observational trial. Clin. Infect. Dis..

[6] Kubo R., Iwanaga N., Nagaoka K., Murai Y., Ashizawa N., Yoshida M., Takeda K., Ide S., Takazono T., Kosai K. (2026). Clinical emergence of *Bacteroides*-associated anaerobic bacteremia due to drug-resistant non-*Bacteroides fragilis* species: A multicentric retrospective observational study. Open Forum Infect. Dis..

[7] Kato H., Hagihara M., Asai N., Mikamo H., Iwamoto T. (2025). A retrospective study comparing the effectiveness of carbapenems and tazobactam/piperacillin as an empirical treatment for patients infected with *Bacteroides fragilis*. Anaerobe.

[8] Wang Y., Guo B., Gao X., Wen J., Wang Z., Wang J. (2023). High prevalence of *cfiA*-positive *Bacteroides fragilis* isolates collected at a teaching hospital in Hohhot, China. Anaerobe.

[9] Jean S., Wallace M.J., Dantas G., Burnham C.D. (2022). Time for some group therapy: Update on identification, antimicrobial resistance, taxonomy, and clinical significance of the *Bacteroides fragilis* group. J. Clin. Microbiol..

[10] Nagy E., Urbán E., Nord C.E., ESCMID Study Group on Antimicrobial Resistance in Anaerobic Bacteria (2011). Antimicrobial susceptibility of *Bacteroides fragilis* group isolates in Europe: 20 years of experience. Clin. Microbiol. Infect..

[11] Gao Q., Wu S., Xu T., Zhao X., Huang H., Hu F. (2019). Emergence of carbapenem resistance in *Bacteroides fragilis* in China. Int. J. Antimicrob. Agents.

[12] Podglajen I., Breuil J., Rohaut A., Monsempes C., Collatz E. (2001). Multiple mobile promoter regions for the rare carbapenem resistance gene of *Bacteroides fragilis*. J. Bacteriol..

[13] Schwensen S.A., Acar Z., Sydenham T.V., Johansson Å.C., Justesen U.S. (2017). Phenotypic detection of the *cfiA* metallo-β-lactamase in *Bacteroides fragilis* with the meropenem-EDTA double-ended Etest and the ROSCO KPC/MBL Confirm Kit. J. Antimicrob. Chemother..

[14] Sóki J., Edwards R., Hedberg M., Fang H., Nagy E., Nord C.E., ESCMID Study Group on Antimicrobial Resistance in Anaerobic Bacteria (2006). Examination of *cfiA*-mediated carbapenem resistance in *Bacteroides fragilis* strains from a European antibiotic susceptibility survey. Int. J. Antimicrob. Agents.

[15] Roh K.H., Kim S., Kim C.K., Yum J.H., Kim M.S., Yong D., Jeong S.H., Lee K., Kim J.M., Chong Y. (2010). New *cfiA* variant and novel insertion sequence elements in carbapenem-resistant *Bacteroides fragilis* isolates from Korea. Diagn. Microbiol. Infect. Dis..

[16] European Committee on Antimicrobial Susceptibility Testing Disk Diffusion Methodology for Anaerobic Bacteria. https://www.eucast.org/ast_of_bacteria/disk_diffusion_methodology.

[17] Nagy E., Becker S., Sóki J., Urbán E., Kostrzewa M. (2011). Differentiation of division I (*cfiA*-negative) and division II (*cfiA*-positive) *Bacteroides fragilis* strains by matrix-assisted laser desorption/ionization time-of-flight mass spectrometry. J. Med. Microbiol..

[18] Wybo I., De Bel A., Soetens O., Echahidi F., Vandoorslaer K., Van Cauwenbergh M., Piérard D. (2011). Differentiation of *cfiA*-negative and *cfiA*-positive *Bacteroides fragilis* isolates by matrix-assisted laser desorption ionization-time of flight mass spectrometry. J. Clin. Microbiol..

[19] Eberly A.R., Wallace M.A., Shannon S., Heitman A.K., Schuetz A.N., Burnham C.D., Jean S. (2022). Development and validation of a novel anaerobic carbapenem inactivation method (Ana-CIM) for the detection of carbapenemase production in *Bacteroides fragilis*. J. Clin. Microbiol..

[20] Wallace M.J., Jean S., Wallace M.A., Burnham C.D., Dantas G. (2022). Comparative genomics of *Bacteroides fragilis* group isolates reveals species-dependent resistance mechanisms and validates clinical tools for resistance prediction. mBio.

[21] Cao H., Liu M.C., Tong M.K., Jiang S., Chow K.H., To K.K., Tse C.W., Ho P.L. (2022). Comprehensive investigation of antibiotic resistance gene content in *cfiA*-harboring *Bacteroides fragilis* isolates of human and animal origins by whole-genome sequencing. Int. J. Med. Microbiol..

[22] Lee Y., Bae M.H., Lee H., Kim M., Lee K. (2025). Evaluation of the disk diffusion test for *Bacteroides fragilis* group clinical isolates. Ann. Lab. Med..

[23] Clinical and Laboratory Standards Institute (2012). Methods for Antimicrobial Susceptibility Testing of Anaerobic Bacteria.

